# Resistance Management for Cancer: Lessons from Farmers

**DOI:** 10.1158/0008-5472.CAN-23-3374

**Published:** 2024-10-02

**Authors:** Sareh Seyedi, Valerie K. Harris, Stefania E. Kapsetaki, Shrinath Narayanan, Daniel Saha, Zachary Compton, Rezvan Yousefi, Alexander May, Efe Fakir, Amy M. Boddy, Marco Gerlinger, Christina Wu, Lida Mina, Silvie Huijben, Dawn H. Gouge, Luis Cisneros, Peter C. Ellsworth, Carlo C. Maley

**Affiliations:** 1 Arizona Cancer Evolution Center, Arizona State University, Tempe, Arizona.; 2 Center for Biocomputing, Security and Society, Biodesign Institute, Arizona State University, Tempe, Arizona.; 3 School of Life Sciences, Arizona State University, Tempe, Arizona.; 4 Department of Ecology and Evolution, University of Lausanne, Lausanne, Switzerland.; 5 University of Arizona Cancer Center, University of Arizona College of Medicine, Tucson, Arizona.; 6 The Polytechnic School, Ira A. Fulton Schools of Engineering, Arizona State University, Tempe, Arizona.; 7 Research Casting International, Quinte West, Ontario, Canada.; 8 Istanbul University Cerrahpasa School of Medicine, Istanbul, Turkey.; 9 Exotic Species Cancer Research Alliance, North Carolina State University, Raleigh, North Carolina.; 10 Department of Anthropology, University of California Santa Barbara, Santa Barbara, California.; 11 Translational Oncogenomics Laboratory, Centre for Evolution and Cancer, The Institute of Cancer Research, London, United Kingdom.; 12 Gastrointestinal Cancer Unit, The Royal Marsden Hospital, London, United Kingdom.; 13 Division of Hematology and Medical Oncology, Department of Medicine, Mayo Clinic, Phoenix, Arizona.; 14 Mayo Clinic, Phoenix, Arizona.; 15 Center for Evolution and Medicine, Arizona State University, Tempe, Arizona.; 16 Department of Entomology, University of Arizona, Tucson, Arizona.

## Abstract

One of the main reasons we have not been able to cure cancers is that treatments select for drug-resistant cells. Pest managers face similar challenges with pesticides selecting for pesticide-resistant insects, resulting in similar mechanisms of resistance. Pest managers have developed 10 principles that could be translated to controlling cancers: (i) prevent onset, (ii) monitor continuously, (iii) identify thresholds below which there will be no intervention, (iv) change interventions in response to burden, (v) preferentially select nonchemical control methods, (vi) use target-specific drugs, (vii) use the lowest effective dose, (viii) reduce cross-resistance, (ix) evaluate success based on long-term management, and (x) forecast growth and response. These principles are general to all cancers and cancer drugs and so could be employed broadly to improve oncology. Here, we review the parallel difficulties in controlling drug resistance in pests and cancer cells. We show how the principles of resistance management in pests might be applied to cancer. Integrated pest management inspired the development of adaptive therapy in oncology to increase progression-free survival and quality of life in patients with cancers where cures are unlikely. These pest management principles have the potential to inform clinical trial design.

## Introduction

Oncologists and pest managers have similar problems in that both cancer cells and pest populations are composed of large numbers of genetically diverse organisms spread over heterogeneous (micro)environments (1–3). When pest managers apply a pesticide to kill pests and oncologists apply a drug to kill cancer cells, they kill the sensitive pests and cancer cells but select for variants that are resistant to the drug (4, 5). In fact, most deaths from cancer are caused by the evolution of therapeutic resistance (6, 7). Both fields also face the challenge of limiting collateral damage and other negative side effects due to the toxicities of their drugs (8, 9). Pesticides have toxic effects on nonpest organisms in the environment and pose a threat to human consumers of the crops, analogous to the toxic effects of anticancer drugs on normal (noncancer) cells and the health of the patient (10, 11).

To date, almost all cancer therapies select for therapeutic resistance (7). Failure to address the evolutionary dynamics of cancer results in the sustained high mortality of metastatic disease and doom future cancer therapies to the same fate. Decades of experimentation in pest management have led to a series of insights and effective heuristics for controlling pests, called integrated pest management (IPM) that, for the most part, have never been tried in oncology. What can oncologists learn from pest managers and how might we translate those insights to the clinic?

IPM is a comprehensive approach to pest management emphasizing multifaceted, system-based strategies involving multiple disciplines (8, 12–15). IPM was initially introduced as “integrated control” in 1959 by Stern and colleagues (16), who synthesized aspects of biological, spatial, and chemical control tactics to reduce pest populations below levels of economic concern while limiting the development of resistance within agricultural pest communities.

Translated to oncology, an analogous approach would be to control cancers so that patients can live with the disease, but not die from it. Although this might seem like a radical change in how we treat cancer, it is already the *de facto* goal in many of the treatments of metastatic cancers in which cures are extremely unlikely (17, 18). The decision of whether to treat with intent to cure or treat with intent to control should depend on evidence for the curability of the cancer as well as the age of the patient, their frailty, and the potential morbidities associated with the different kinds of treatment. Control would likely be more palatable in an elderly patient compared with a pediatric patient.

Here, we briefly review the history of the emergence of acquired therapeutic resistance and early attempts to address it by using multiple drugs in both pest management and oncology. We then review the principles of IPM (19) and discuss how they might be translated to oncology. Finally, we review early promising results in the application of those principles to oncology and provide an example of a potential future clinical trial in colorectal cancer based on IPM.

## Single-Drug Therapies Tend to Fail

As early as 1914, pest managers observed that single-agent pesticide treatments did not work long-term because they selected for pesticide resistance (10). New insecticides developed in the 1960s and 70s were initially highly effective but quickly lost their effectiveness due to insects evolving mechanisms of resistance (20–22). Insects even became resistant to alternative methods of control like crop rotation (23, 24).

Acquired therapeutic resistance was also documented in the first clinical reports of chemotherapy for cancer (25, 26). Single-drug therapies, such as nitrogen mustard and aminopterin, were used in the treatment of tumors in the late 1940s (25, 26). However, acquired resistance to treatment was observed for both drugs (25, 26). Resistance also evolved in response to radiotherapy (27). This helped drive interest in developing chemotherapies as the main method to treat cancer (25).

Targeted therapies, including both small molecules and biologics, also select for resistance (7, 28). Perhaps the most famous targeted therapy, imatinib mesylate, eventually leads to resistance in chronic myelogenous leukemia, gastrointestinal stromal tumors, and in Philadelphia chromosome–positive acute lymphoblastic leukemia (29). Trastuzumab, targeting HER2^+^ breast cancer, only worked for a median of 9 months in one study (30) and 4.9 months in another study, with the majority of patients developing resistance within a year (31). Targeted therapies for EGFR are notorious for selecting for resistance mutations. Most patients with non–small cell lung cancer (NSCLC) relapse on gefitinib (32), erlotinib (33), osimertinib (34), or crizotinib (35).

Recently immunotherapies, in particular immune checkpoint blockade therapies, have received well-deserved attention and excitement for generating responses, and sometimes apparent cures in some of the most difficult kinds of cancer to treat: lung cancers and melanoma. However, they also often select for acquired therapeutic resistance (36–38). In melanoma, between a quarter to a half of patients acquire resistance and progress within 5 years on immune checkpoint blockade therapy (36–40), and as many as 65% of patients with NSCLC progress within 4 years (41).

## Multidrug Therapies Tend to Fail

Multidrug therapies are often better than single drugs but still tend to fail, particularly in late-stage disease (42–47). In theory, if resistance to a single drug is rare in a population of pests or cancer cells and the mechanism of resistance is different for each drug, resistance to multiple independent drugs should be much rarer than resistance to a single drug. Experience has shown that this is not the case. Even though several studies have shown that combined therapy is better than monotherapy in both pest management and cancer therapy, combined therapy still selects for multidrug resistance and ultimately fails (42–48). This is in part due to cross-resistance between drugs and mechanisms of multidrug resistance, such as the upregulation of efflux pumps that can provide resistance to drugs with independent modes of action (MoA; refs. 45, 49, 50). Therefore, in most cases, combining drugs has not solved the problem of acquired therapeutic resistance.

### Evidence in pest management

In 1989, Tabashnik provided a detailed review of the failures of multiple drug usage in pest management (5). The combinatorial use of insecticides in several studies showed that this method could not prevent resistance significantly (42–44, 51–53). There is an extensive and old literature in pest management that discusses different ways to calculate and detect synergy (and antagonism) between drugs, most of which depend on generating a model of the expected effect of the combination and comparing it to the observed effect (54). Plana and colleagues (55) have applied similar methods for defining (and finding very little) synergy in cancer drug combinations. There are important concerns about the combinatorial use of drugs, including toxicity for the natural predators of the pests, development of resistance in secondary pests, and increased selective pressure for cross-resistance (5).

Ozaki and colleagues (42) combined pesticides with potentially synergistic effects and different MoAs. They found that combinations of two or more insecticides either together, or alternating single drugs could delay the development of resistance, though multi-insecticide resistance still evolved (42). MacDonald and colleagues (44) tested the use of single insecticides to control house flies and also used multiple drugs in rotation. Overall, resistance most rapidly emerged in the single-drug treatment groups. Alternating drugs reduced the evolution of resistance and delayed resistance in the field (44). In practice, even theoretically synergistic combinations of pesticides are selected for multidrug resistance (14).

Overall, even though combining insecticides remains a theoretically justified tactic for delaying resistance in the case of pest management, mixtures that meet the optimization criteria of models remain elusive in practice (52).

### Evidence in late-stage cancer therapy

Early-stage cancer is often curable through resection, and survival rates are typically much higher than late-stage cancers (56). The standard of care in many late-stage cancer treatments is to combine drugs (50). It is widely acknowledged that single-drug therapies tend to fail in cancer. What is less widely acknowledged is that multidrug therapies also tend to fail, particularly in late-stage adult cancers, due to multidrug resistance (45). Meta-analyses of lung (50, 57–59), breast (60), gastric (60, 61), pancreatic (62–64), and ovarian cancers (65, 66) show modest survival benefits of combining drugs with increased toxicity but rarely cures from complex combined therapies.

A meta-analysis of 35 randomized phase III trials in NSCLC compared single-drug therapy versus two or three drugs (50). There was a significant increase in both tumor response [OR, 0.42; 95% confidence interval (CI), 0.37–0.47; *P* < 0.001] and 1-year survival (OR, 0.80; 95% CI, 0.70–0.91; *P* < 0.001) using doublet regimes compared with single-drug therapy. In addition, they found an increase in the tumor response rate when three drugs were used in combination (OR, 0.66; 95% CI, 0.58–0.75; *P* < 0.001). However, there was no increase in 1-year survival when the third drug was added (OR, 1.01; 95% CI, 0.85–1.21; *P* = 0.88). Overall, greater toxicity was also observed in combination chemotherapy compared with single-agent chemotherapy (50). Grade 3 and 4 toxicity rates were higher with doublet regimens compared with single-agent therapy (ORs 1.2–6.2), as well as in triplet regimens compared with doublet regimens (ORs 1.4–2.9; ref. 50). A previous meta-analysis of single versus combination therapy for NSCLC found a 22% increase in 1-year survival with combination therapy compared with single-agent chemotherapy (RR: 1.22; 95% CI, 1.03–1.45), but a 3.6-fold increase in the risk of treatment-related death (RR: 3.5; 95% CI, 1.8–6.7; ref. 59).

A later randomized phase III trial compared paclitaxel alone versus paclitaxel and carboplatin in advanced NSCLC and found no statistically significant improvement in overall survival (OS) in the combined therapy (57). The 1-year survival was 32% and 37% for single and combination therapy, respectively, with a HR of 0.91 (95% CI, 0.77–1.17; *P* = 0.25; ref. 57). Hematologic toxicity and nausea were more frequent in the combination arm, but febrile neutropenia and toxic deaths were equally low in both arms.

A more recent meta-analysis comparing combined chemotherapy as second-line therapy with single-agent chemotherapy in the treatment of patients with advanced NSCLC from phase II and III clinical trials found no significant difference in OS between the combined and single-agent therapy (*P* = 0.32). However, doublet chemotherapy caused more grade 3 to 4 hematologic (41% vs. 25%; *P* = 0.0001) and nonhematologic toxicities (28% vs. 22%; *P* = 0.034; ref. 67).

In breast cancer, a meta-analysis of 43 randomized controlled trials included 9,742 patients who received chemotherapy in combination or a single agent as their first-line of treatment (60). They found that combined therapy significantly improved OS and time to progression (HR, 0.88, 95% CI, 0.83–0.93, *P* < 0.00001) compared with single-agent therapy but only delayed time to progression by a matter of months (60). Furthermore, women who received combined therapy faced serious side effects, such as vomiting and nausea, due to the toxicity of the treatment (60). In general, combining chemotherapy treatments showed a notable correlation with higher rates of leukopenia (OR 1.45; 95% CI, 1.28–1.65; *P* < 0.00001), alopecia (OR 1.55; 95% CI, 1.32–1.81; *P* < 0.00001), and nausea and vomiting (OR 1.65; 95% CI, 1.41–1.93; *P* < 0.00001), compared with single-agent treatment (60).

A meta-analysis of first-line chemotherapy in advanced gastric cancer on randomized phase II and III trials found that combined drug therapy (two or three drugs) improved survival (HR, 0.83; 95% CI, 0.74–0.93) compared with single-drug therapy. However, toxicities related to the treatment were higher in combined strategies (68). A study comparing the combination of three drugs (docetaxel, cisplatin, and fluorouracil or DCF) versus two drugs (cisplatin and fluorouracilor or CF) in phase III as first-line therapy of advanced gastric cancer found that OS (*P* = 0.02) and time to progression (*P* < 0.001) were longer for three drugs compared with two drugs. In addition, the 2-year survival rate on three drugs was 18% versus 9% for two drugs, led to higher toxicity (69) and clearly did not solve the problem of therapeutic resistance. However, grade 3 to 4 treatment-related adverse events were more frequent in the DCF group (69% vs. 59% in the CF group), particularly complicated neutropenia (29% in DCF vs. 12% in CF; ref. 69).

Meta-analyses of phase II and III clinical trials in pancreatic cancer have shown an advantage in using multidrug therapies, with improvements in tumor response and patient survival, but cures were still rare, and combination therapies increased toxicities compared with single-drug therapies. Heinemann and colleagues (63) analyzed patients with advanced or metastatic pancreatic cancer and found that combining gemcitabine along with another cytotoxic drug versus gemcitabine alone produced a small but significant improvement in OS (HR: 0.91; 95% CI, 0.85–0.97; *P* = 0.004). However, there is significantly more grade 3 to 4 toxicity in the combined regimens (70). This may help explain why patients with good performance status (PS) had a notable survival advantage with combination chemotherapy (HR, 0.76; *P* < 0.0001), whereas those with poor PS did not benefit (HR, 1.08; *P* = 0.40; ref. 63).

Another study by Moore and colleagues (71) on 569 patients of randomized phase III trials with advanced pancreatic cancer showed both OS (HR, 0.82; *P* = 0.083; 95% CI, 0.69–0.99) and 1-year survival (23% vs. 17%, *P* = 0.023) improvements using a combination of gemcitabine with erlotinib compared with using gemcitabine alone. Progression-free survival was also significantly better in this combination compared with single-agent therapy (HR, 0.77; 95% CI, 0.64–0.92; *P* = 0.004). However, there were few cures, and adding erlotinib to gemcitabine was associated with more grade 1 or 2 toxicity (71).

Pusceddu and colleagues (72) conducted a meta-analysis comparing the efficacy of two chemotherapy regimens in treating patients with metastatic pancreatic cancer: gemcitabine with nab-paclitaxel versus FOLFIRINOX (5-fluorouracil, oxaliplatin, and irinotecan). Neither multidrug therapy reliably achieved cures, and there was no difference in the OS between the 2- and 3-drug regimens (HR, 0.99; *P* = 0.9; 95% CI, 0.84–1.16). Both regimens were associated with grade 3 and 4 toxicities, with more anemia and neurotoxicity in gemcitabine with nab-paclitaxel and more neutropenia in FOLFIRINOX (72).

A meta-analysis of four randomized II and III trials with a total of 1,300 patients with ovarian cancer comparing single-agent platinum chemotherapy versus platinum combined with another chemotherapy (e.g., carboplatin with gemcitabine), in women with relapsed platinum-sensitive ovarian cancer, found an improvement in OS when using combined platinum chemotherapy (HR, 0.80; 95% CI, 0.64–1.00; *P* = 0.05; ref. 65). Two of the four trials included quality of life data, which showed no adverse effect on the quality of life between the single and combined platinum treatments (65). The 3-year OS when using carboplatin alone was 29% compared with 42% when using both carboplatin and epidoxorubicin (OR = 0.8; 95% CI, 0.6–1.2). However, platinum combined therapy led to higher toxicity (65).

Multidrug therapies that include immunotherapies and/or targeted therapies also tend to fail in late-stage cancers, showing that acquired therapeutic resistance is not just a problem for chemotherapies. Combining immune checkpoint inhibitors has shown significant improvements in OS across various malignancies, yet long-lasting survival is achieved by only 20% to 40% of patients (73), and increased toxicity is common (74–76). Dual immune checkpoint blockade with anti–PD-1 and anti–CTLA-4 has resulted in high response rates (58%) in patients with metastatic melanoma, but nearly half of them experienced severe treatment-related side effects, with uncertain long-term survival benefits (75, 76).

Adding an immune checkpoint inhibitor to chemotherapy has shown some evidence of minor improvements in OS but also increased toxicity and little evidence of cures in triple-negative breast cancer (77, 78) and head and neck squamous cell carcinoma (79), as well as gastric, gastro-esophageal, and esophageal cancer (80–82). Similarly, adding targeted therapy to chemotherapy produced a minor improvement in survival along with an increase in grade 3 to 4 toxicity in a meta-analysis of gastric cancer (83), although it did not improve OS in meta-analyses of NSCLC (84) or triple-negative breast cancer (85).

In all these studies and meta-analyses, investigators found evidence of increased efficacy for multiple drugs over single drugs, often at the cost of increased toxicity and treatment-related deaths. However, we must face the fact that whereas multidrug therapy may extend life by months, it rarely achieves a cure in late-stage disease, and there are diminishing returns from adding more drugs due to increases in toxicity and the evolution of multidrug resistance. In other words, multidrug therapy does not solve the problems of cancer or pest infestations.

## Mechanisms of Resistance

Insects have evolved resistance to pesticides through a variety of mechanisms that are familiar to cancer biologists. These include modifications to the drug target, metabolic detoxification, reduced activation of the drug, prevention of drug uptake, and cellular efflux pumps. Insects often evolve resistance through changes in the structure of the target molecule, called “target site insensitivity.” These mutations generally reduce the binding affinity of the drug for the target molecule (86–89). Drug target modifications are often observed in cancer, including the well-known T790M mutation in EGFR accounting for more than 50% to 60% of the cases of resistance to erlotinib therapy in lung adenocarcinoma (90, 91). Both pests and cancer cells can evolve resistance through amplification or overexpression of the target gene (92, 93). Another common mechanism of resistance to pesticides is metabolic detoxification through cytochrome P450s, esterases, and glutathione S-transferases (88), which have also been observed in cancers (94, 95). In some cases, resistance to a drug or a prodrug may evolve via inactivation or downregulation of the enzyme that would have activated the drug. This is seen in both proinsecticide resistance (96–98) and cancer prodrug resistance (86, 87). There is (less common) evidence of resistance through prevention of uptake of drugs by cancer cells (99, 100) and pests (101, 102). Finally, cellular efflux pumps, such as major facilitator superfamily and ATP binding cassette transporters, are often implicated in pesticide resistance (101, 103–105) as well as cancer drug resistance (86, 87, 99, 106) and in both cases can generate multidrug resistance, including cross-resistance between chemotherapies, radiotherapy, and immunotherapy (107). Like cancer, in some cases, pesticides trigger upregulation of resistance phenotypes like detoxification enzymes and efflux pumps, but can also be mutagens, causing *de novo* resistance mutations (108).

Some mechanisms of resistance seem to be unique to either pest or cancer cell resistance. In some cases, insects evolve resistance to pesticides through behavioral or physiologic changes, such as changes to the cuticle that prevent penetration of the drug or excretion of the drug from the insect’s body, that do not have a direct parallel in cancer cells (109, 110). Conversely, cancers often evolve resistance to drugs through activating pathways that compensate for a drug’s effect, such as an alternative proliferative signal, DNA repair to compensate for DNA damaging drugs (90, 91, 111, 112), or evolving alternative methods of evading the immune system under immunotherapy (113–117). Cancers also disable apoptotic pathways and thereby prevent drug-induced cell death (99, 100, 111, 118). Cancers have also been shown to upregulate a gene (MUC4) that binds to the target of a drug (HER2) and out-competes the drug (trastuzumab) while activating the HER2 receptor (30). The absence of reports of these forms of drug resistance for pesticides may be due to the fact that most pesticides do not work through shutting down a proliferation signaling pathway or triggering apoptosis but achieve lethality through other mechanisms such as neurotoxicity (92). There are exceptions. Methoprene and methoxyfenozide seem to kill insects through inhibition of cellular proliferation (119), and azadirachtin A induces cell-cycle arrest and apoptosis through p53 activation (120); however, pest resistance seems to evolve through detoxification, not pathway compensation or disabling apoptosis (121–124).

## Modern Principles of IPM

**Figure 1.**
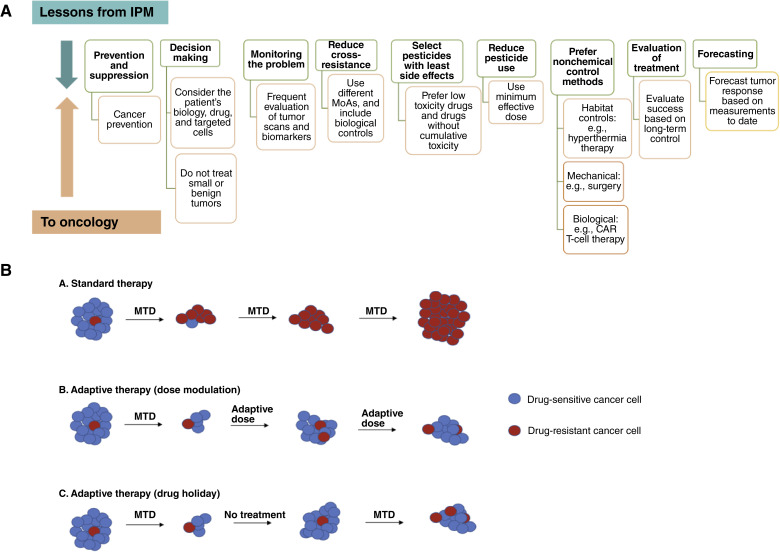
Lessons from IPM to oncology. **A,** These are the best practices in pest management and how they might be applied in cancer treatment. **B,** Comparison of standard therapy with two models of adaptive therapy. In standard therapy (A), the MTD of a drug is used. In dose modulation adaptive therapy (B), the dose of the drugs is adjusted based on the tumor’s response. In drug holiday (C) treatment skipping, the MTD of the drug is used until the tumor shrinks and then the treatment is skipped. CAR, chimeric antigen receptor.

Through decades of experimentation and observation, pest managers have learned to focus on management, rather than eradication of pests (13). They have developed a set of principles for maintaining long-term management of pest populations (Fig. 1A; refs. 8, 19):

- Prevention and suppression: adjust crops and the habitat to suppress the proliferation of pests and favor crop production.
- Monitor continuously using systems for early warnings.
- Decision-making: identify acceptable thresholds of infestation below which there will be no intervention and change interventions in response to changes in pest levels.
- Prefer nonchemical control methods including mechanical, biological, and habitat controls.
- Use pesticides as specific as possible to the type of pest, minimizing off-target effects, including pesticides that modify pest behavior rather than kill the pests.
- Use the lowest effective dose possible.
- Choose pesticides to reduce cross-resistance and separate their application in space and time.
- Evaluate success based on long-term management.
- Forecast pest growth and response to interventions to inform most of the other principles.

Pest managers are aware that no single intervention is sufficient to control pests, and that usually the use of several chemicals is necessary (8). However, combining these principles and combining chemical controls with other forms of controls can lead to the effective and long-term management of pests (8, 14, 15). Hence, in the term integrated pest management, the emphasis is on both integration and management.

The principles of IPM may be applied to any type of cancer. Here, we describe how these principles might be employed in the design of a clinical trial for colorectal cancer as a concrete example to illustrate these ideas. Of course, before such a trial is opened, the innovations from IPM that have not yet been tested in animal models should be studied in preclinical models. We chose colorectal cancer because (i) it is common (125); (ii) it is difficult to manage the late stages of this disease (126); and (iii) there is a variety of drugs that can produce an initial response (127), and so could be rotated, or adjusted in an IPM-inspired protocol. Inclusion criteria for such a clinical trial should prioritize stage III and IV patients that have not received prior chemotherapeutic interventions, or for which only one MoA has failed and where complete resection is either not possible or was unsuccessful. The primary outcome from this study should be OS, with secondary outcomes including measures of quality of life and time to progression (defined as the tumor continuing to grow despite the use of all available drugs). The goal here is to manage cancer as a chronic disease so that patients can live with their cancers but not die from them, or from the treatments.

### Prevention and suppression

Cancer biologists, like pest managers, have noted that prevention should be a key part in the strategy of dealing with cancer (1, 3, 10, 128), well before the point of a therapeutic clinical trial. It is much easier to prevent an infestation or cancer than it is to treat it. Translating these ideas to oncology might involve modifying the microenvironment of a benign tumor to favor healthy cells and disfavor malignant cells (129), which we discuss below under habitat controls. Other strategies, such as improving the overall health of patients through diet and exercise, and other lifestyle factors are familiar and could be helpful in making the tissue microenvironments less favorable for cancer (130).

### Decision-making (consider the patient’s biology, drug, and target cells)

An ideal trial based on IPM should start with extensive sampling and profiling the patient’s cancer to determine what known forms of resistance are present in the tumor as well as what targetable mutations are present and at what frequencies, as well as whether the tumor is growing. Pest managers would not even treat a field if the infestation is below acceptable levels of crop destruction. Translated to oncology, this would suggest watchful waiting as long as the tumor is small enough and there are no signs of significant damage to the patient. The principle is to establish a threshold below which we do not treat. These thresholds may be based on multiple objectives such as quality of life, risk of progression, PS, and other measures of health. We do not currently know what the tradeoffs will be between starting therapy early and selecting for resistance early versus living with some low tumor burden and the potential damage from that (131). The specific thresholds to be used will have to be determined by theoretical, preclinical, and clinical experiments. Withholding therapy does not imply missing the opportunity to cure the patient. Rather, withholding therapy preserves the opportunity to use drugs effectively in the future to control the tumor.

Decision-making should take into account and exploit aspects of the pest (and cancer cell) biology. For example, Lygus bugs, which damage cotton, lay their eggs in the height of summer. They take 1 week to hatch into nymphs and another week to develop to the point that they begin to damage crops. Efficacy of control is optimized by timing and targeting for nymphs. Pest managers exploit this synchronized development with a 2-week window to detect and treat the crops. Similarly, cancer therapy efficacy can be enhanced through careful timing of drug application, either by timing chemotherapy to circadian cycles so as to protect normal tissues (132) or by attempts to synchronize cancer cells in order to sensitize them to therapy (133–135).

### Monitor regularly and change interventions in response

It is unclear how often a tumor should be monitored during treatment as this will vary between patients, tumor types, and interventions. It may even vary temporally within a given patient as the dynamics of their cancer slow down or speed up. Traditionally, oncologists do not monitor tumor burden during a therapy protocol and only check weeks to months later to determine whether the therapy is effective. For example, in a clinical study that was conducted on patients with rectal adenocarcinoma, their response to chemoradiation was determined only 2 to 4 weeks after the treatment was completed (136). In cases in which a solid tumor is monitored during therapy, RECIST guidelines are to evaluate it every 6 to 8 weeks (137), although evaluation every 12 weeks is common (138). Monitoring tumor response intensively during treatment allows for adjusting to its response.

Adaptive therapy is one example of the application of pest management to oncology, based on monitoring and adjusting therapy in response to tumor dynamics. Adaptive therapy seeks to prevent the expansion of therapeutically resistant clones by maintaining chemosensitive cells in the tumors to compete with the resistant cells (45, 46, 139, 140). This is done operationally by trying to keep the tumor at a stable size. In the dose modulation version of adaptive therapy, the tumor burden is measured frequently and the dose is increased if the tumor is growing, but the dose is lowered if it is shrinking. Otherwise, if the tumor is stable, dosing is kept at the same level (Fig. 1B). This dramatically extended time to progression and survival in mouse models of ovarian and breast cancer (45, 139, 141). The amount of drug required to keep the tumor stable also decreased over time, possibly because tumors in the adaptive therapy arms evolved better perfusion than the tumors in the MTD arms (45, 139). The only clinical trial of adaptive therapy that has completed to date used a different protocol, similar to intermittent therapy, in which dosing stopped when the tumor dropped below 50% of its initial burden, and was restarted when it recovered to the level of the initial burden. This resulted in the doubling of radiographic progression-free survival in castration-resistant metastatic prostate cancer (47). Dose adjustment works better than intermittent adaptive therapy in both computational and mouse models (45, 139, 142). In a colorectal cancer clinical trial, we would propose using dose modulation adaptive therapy with each single drug until the tumor grows and then rotating to a new drug with a different MoA.

### Prefer nonchemical control methods

One way to prevent the evolution of therapeutic resistance to drugs is to use nonchemical forms of control. Pest managers distinguish three types of nonchemical controls: mechanical, habitat, and biological.

#### Mechanical controls

Pest burden can be reduced by physically removing pests, erecting barriers to their entry, and setting up traps to contain them. Using “lure and kill” traps has been shown to be an effective supplemental strategy in a variety of pest management studies (143). Surgery is a common technique for physically removing cancer cells (144). Removing the primary tumor and metastases reduces the size of the cancer cell population and thereby reduces the evolvability of cancer. This should be beneficial even if not all metastases can be removed (145). In fact, mechanical removal of tumors (cytoreductive surgery) has been shown to increase survival in many cases of metastatic cancer, where the tumor burden can be substantially reduced (145, 146). This was true even for recurrent cancer (147). However, the benefits must be weighed against the morbidity and risks of the surgery. Cancer traps, potentially including chemoattractants for cancer cells, might complement other control mechanisms and help prevent further metastases (148, 149).

#### Habitat controls

Translated to oncology, using habitat controls would mean making the microenvironment unfavorable to the cancer cells and favorable to the normal cells as much as possible. In some cases, we might be able to exploit the loss of functions that may accumulate in cancers. Fasting may cause normal cells to become quiescent because they respond to growth inhibition signals whereas cancer cells likely continue to proliferate. This can protect normal cells from the toxic effects of cell cycle–specific drugs (150). Antiangiogenic drugs are designed to make the microenvironment less hospitable for cancers, although they have had mixed success (151).

#### Biological controls

Biological control in cancer treatment includes immunotherapy (predators) as well as viral and bacterial therapy. Engaging predation from the immune system may include cancer vaccines (152), immune checkpoint modulators (153), and chimeric antigen receptor T-cell therapy (154, 155).

### Use drugs with specificity

Using drugs specific to a cancer helps to minimize off-target effects. Cancer cells are derived from human cells, so it is inherently difficult to develop cancer-specific therapies that do not affect normal cells. If sequencing or other profiling of cancer identifies clonal mutations that may be targeted by a drug, they should be included in the rotation of therapies along with the more broad-spectrum therapies.

### Use the lowest dose possible

Most chemotherapeutic drugs can be characterized by a dose–response curve; the higher the drug dose delivered, the more cancer cells are killed (156, 157). This relationship between dose and cell death is limited at high doses by diminishing returns as well as unacceptable toxicity (156, 157). This relationship naturally led to the use of the MTD in most cancer therapy protocols (158, 159). For drugs for which toxicity is not limiting, like some hormone and targeted therapies, researchers have used the minimum dose that achieves the maximum biological efficacy, called the optimal biological dose (OBD; ref. 160). There is no consensus as to how “efficacy” should be defined in the OBD. If it is defined by tumor shrinkage, then this will lead to very high selective pressures on the cancer cells (160, 161). Unfortunately, we know from evolutionary theory that using the maximum tolerated (or effective) dose is also the fastest way to select for acquired therapeutic resistance (140). The strength of selection is directly related to the speed of evolution. This implies that we should be using the minimum dose necessary to control the tumor in order to minimize the selective pressure for resistance. Simulation studies bear this out (142). Dose modulation adaptive therapy is a protocol for finding that dose (45, 46, 139, 140). Note that if efficacy is defined as OS time of the patients, then the OBD may well be something close to the minimum dose necessary to control the tumor.

### Reduce cross-resistance

Pest managers have learned to reduce cross-resistance by diversifying the MoA of drugs they use and partitioning those drugs in space and time as much as possible (8, 13–15).

#### Diversify the use of MoAs as much as possible

Cancer drugs may be grouped by the MoA they use to achieve their anticancer effects. Both theory and experiment show that resistance to one drug with a particular MoA also confers cross-resistance to other drugs that use the same MoA (162, 163). Thus, to avoid cross-resistance and reap the benefits of using multiple drugs, those drugs should be chosen so that they use different MoAs (19). This principle has been recognized in oncology, although it is not always followed (164, 165). We might be able to do even better in improving patients’ survival by combining drugs for which resistance to one drug causes sensitivity to the other, and vice versa, an approach called “double-bind therapy” in cancer (19, 128, 166, 167) and “negative cross-resistance” in pest management, in which cytochrome P450s detoxify some pesticides but activate other pesticide prodrugs (168).

#### Partition MoAs in space or time to segregate their usage

As we discussed above, the combination of drugs with different MoAs does not solve the problem of acquired therapeutic resistance, due to phenomena like multidrug resistance in both cancer (106) and pest management (169, 170). So pest managers advise separating the application of different drugs as much as possible in both time and space, including refuges that are not sprayed so as to preserve drug-sensitive pests (14). Although spatial partitioning is difficult in cancer, drugs with rapid half-lives could be used so that one drug is completely cleared from the system before cancer cells are exposed to the next drug (171).

**Figure 2.**
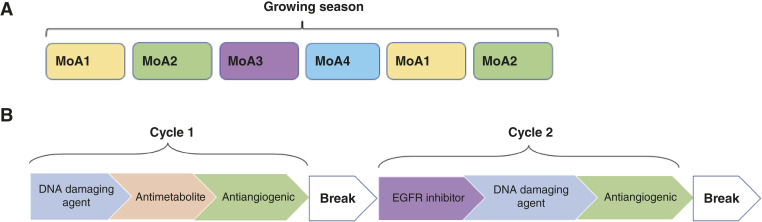
Schematic of a MoA schedule. **A,** In pest management, pest managers avoid sequential use of the same MoA and avoid using a MoA more than twice within the same growing season. To avoid cancer cells acquiring therapeutic resistance, repeated applications of a MoA should be separated as much as possible. **B,** Proposed scheduling of drugs that applies each drug once per 4-week cycle and uses different MoAs. The third week of each cycle uses an antiangiogenic drug to avoid restricting the vasculature before trying to deliver the other drugs. Week 4 of each cycle is a drug holiday break to allow the patient to recover.

Perhaps the most striking difference between oncology and pest management is that pest managers try to never apply the same MoA twice in a row, and never more than twice within a growing season (e.g., the Arizona Cotton IPM strategy; Fig. 2A). In oncology, we repeatedly apply the same drug or drug combinations, week after week. In contrast, pest managers endeavor to switch pesticides every time they spray a field (8). This prevents a resistant clone from expanding much before it is exposed to a new drug with a different MoA. This heuristic of only applying a drug once, or at most twice, leads to a different perspective on decision-making compared with oncology. Pest managers will sometimes delay the application of a drug to maximize its impact if they anticipate a coming influx of pests, even if the current pest level is above the threshold that would normally trigger treatment. This difference from traditional protocols in oncology derives from the goal of control, rather than cure.

We identified existing FDA-approved drugs for colorectal cancer with four distinct MoAs: DNA damaging agents, including DNA synthesis blockades and platinum therapies, antimetabolites, EGFR inhibitors, and VEGF inhibitors. After 3 weeks (with one drug each week), the patients would receive a further week break to complete the 4-week cycle. The third week in each cycle could be an antiangiogenic drug (to restrict the tumor vasculature and nutrients once the other drugs have been delivered; Fig. 2B). This restriction of resources should make it especially hard for resistant cells to grow in comparison to sensitive cells, because the former often require more resources to pay the fitness cost of resistance (45).

### Evaluation based on long-term control

Prior to the development of IPM, farmers would generally evaluate pest management methods by a combination of crop yields and the absence of pests (8). Practices prior to IPM were usually “womb-to-tomb” spraying, in which pesticides were used until the end of the production cycle (172). Successful management of pest resistance required a shift in evaluation criteria away from short-term response and the impractical goal of total pest eradication to the more helpful goals of long-term management, cumulative yield, and stability of yield over time (8). The parallel for oncologists may be to move away from partial and complete responses as the criteria of evaluation and focus on time to progression, survival time, and quality of life.

### Forecasting

Forecasting is central to IPM efforts and it informs much of what pest managers do including decision-making, monitoring, prevention efforts, managing resistance, and evaluation. However, formal forecasting is rarely applied in oncology. Gu and colleagues (173) studied patients with glioblastoma multiforme and demonstrated the impact of a predictive model based on patient-specific planning of the treatment strategy. It is helpful to fit models of sensitive and resistant cell dynamics to tumor burden measures in order to forecast the expected results of continuing the current treatment versus changing treatment (174, 175). The more often a tumor can be monitored, the better those forecasts can be (176).

### Summary of the clinical trial

Each of the innovations in this example clinical trial ought to first be evaluated in preclinical models (141), although those models must demonstrate acquired therapeutic resistance under the standard of care in order to faithfully represent the clinical challenge. Colorectal cancer remains an appealing candidate disease due to the existence of well-described spontaneous murine models (177). If preclinical experiments with the above design are successful, clinical trials may be able to bypass phase I clinical trials due to the fact that safe dosage ranges for the drugs in our design would have already been determined. For phases II and III, patient recruitment should involve those in the second line of treatment and exclude drugs with a similar MoA from the first line of treatment that was used for those patients.

### Challenges and clinical needs for IPM-inspired oncology

In order to develop IPM-inspired treatments for cancer, there are a few important challenges that will need to be addressed. First, it is not clear how frequently we will need to measure that tumor burden to appropriately adjust our interventions. We need economical and safe ways to measure tumor burden in order to monitor cancers frequently. This may require the development of new technologies.

Liquid biopsies (blood, serum, or plasma, as well as urine, cerebrospinal fluid, saliva, and pleural effusion fluids) carry a wealth of information that might be used to monitor tumor burden and response, including circulating tumor cells, ctDNA, circulating tumor RNA (particularly microRNAs), tumor-educated platelets (178, 179), extracellular vesicles (180), and proteins (181, 182). ctDNA in the blood is particularly promising as a minimally invasive biomarker for monitoring cancer (183). It may not only measure tumor burden but also be more generally representative of the cancer cell population and molecular diagnosis (mutations of the tumor) than a single biopsy (183). It may also reveal mutations that cause resistance and thus enable monitoring of some resistant populations as well as overall tumor burden (184–186). ctDNA has been shown to be clinically useful across a variety of domains including diagnosis and early detection, monitoring treatment response, detecting minimal residual disease as well as recurrence, and generally guiding personalized therapy (187).

There are a variety of different assays applied to ctDNA, from targeted panels with high sensitivity (188, 189) to genome wide assays (190) and to methylation (191, 192) or fragment patterns (193), which show promise. However, methods for using ctDNA as a tumor marker are still under development and are not yet standardized for most clinical applications (181, 182, 184, 187, 193–195).

Second, we need biomarkers to help predict if a cancer is curable or if we should focus on management instead. In particular, if we could distinguish cancers that are likely to harbor therapeutically resistant clones from cancers that do not, perhaps through measuring intratumor heterogeneity (196), we could focus on controlling the former and curing the latter.

Finally, many of the ideas coming from IPM would need to be tested in animal models to provide sufficient evidence for their efficacy to justify clinical trials.

## Conclusions

Drug resistance is one of the most important problems we face in clinical oncology. Solving it will likely require the integration of oncology with evolutionary biology, ecology, bioinformatics, and inspiration from other fields involved in managing systems that evolve therapeutic resistance, including pest management, weed management, and infectious diseases (viruses, bacteria, protozoa, fungi, and helminths; refs. 197, 198).

Here, we have shown that pest managers face the same problem as oncologists, with pesticides selecting for resistant pests. In both cases, a single drug tends to fail. Combination therapy with different MoAs is more effective than single-drug therapies but still leads to multidrug resistance after a short period of time. However, IPM dynamically slows the development of pesticide-resistant pests often below densities requiring control over the long term (15). Nonchemical and chemical approaches used in IPM may be translated to cancer therapy. Modern principles of resistance management could control the growth of resistance by limiting the use of each MoA (minimizing the frequency of use and dose to the lowest practical levels), diversifying the use of MoAs through rotation programs, partitioning MoAs use in space or time, and utilizing nonchemical methods of control.

In fact, a general principle underlying most of the lessons of IPM is that the more drugs we use, the more we select for resistance. Work on therapeutic resistance in malaria has come to similar conclusions (199, 200). Most strategies of IPM are ways to limit the amount of drug used, although still controlling the infestation. IPM inspired adaptive therapy in oncology (45, 139, 140). An ideal trial based on IPM would start with intensively monitoring cancer and rotating drugs at minimum effect doses, stopping dosing when possible and avoiding using any one MoA for so long as to select for resistance. If we assume that resistant cells are already present, as pest managers do, this approach represents a fundamental shift from trying to cure cancer to controlling it, with the aim of prolonging patient survival and quality of life. This is a call to carry out the preclinical experiments and clinical trials to test these ideas and generate the evidence for improving clinical practice. By transcending the sole focus on a cure, we open up new possibilities for methods to dramatically improve both patient survival and quality of life.
